# Large‐scale functional network connectivity mediate the associations of gut microbiota with sleep quality and executive functions

**DOI:** 10.1002/hbm.25419

**Published:** 2021-03-19

**Authors:** Huanhuan Cai, Chunli Wang, Yinfeng Qian, Shujun Zhang, Cun Zhang, Wenming Zhao, Tingting Zhang, Biao Zhang, Jingyao Chen, Siyu Liu, Jiajia Zhu, Yongqiang Yu

**Affiliations:** ^1^ Department of Radiology The First Affiliated Hospital of Anhui Medical University Hefei China; ^2^ Research Center of Clinical Medical Imaging, Anhui Province Hefei China; ^3^ Anhui Provincial Institute of Translational Medicine Hefei China; ^4^ Department of Clinical Laboratory The First Affiliated Hospital of Anhui Medical University Hefei China

**Keywords:** executive functions, functional connectivity, functional magnetic resonance imaging, gut microbiota, large‐scale brain networks, sleep quality

## Abstract

Network neuroscience has broadly conceptualized the functions of the brain as complex communication within and between large‐scale neural networks. Nevertheless, whether and how the gut microbiota influence functional network connectivity that in turn impact human behaviors has yet to be determined. We collected fecal samples from 157 healthy young adults and used 16S sequencing to assess gut microbial diversity and enterotypes. Large‐scale inter‐ and intranetwork functional connectivity was measured using a combination of resting‐state functional MRI data and independent component analysis. Sleep quality and core executive functions were also evaluated. Then, we tested for potential associations between gut microbiota, functional network connectivity and behaviors. We found significant associations of gut microbial diversity with internetwork functional connectivity between the executive control, default mode and sensorimotor systems, and intranetwork connectivity of the executive control system. Moreover, some internetwork functional connectivity mediated the relations of microbial diversity with sleep quality, working memory, and attention. In addition, there was a significant effect of enterotypes on intranetwork connectivity of the executive control system, which could mediate the link between enterotypes and executive function. Our findings not only may expand existing biological knowledge of the gut microbiota‐brain‐behavior relationships from the perspective of large‐scale functional network organization, but also may ultimately inform a translational conceptualization of how to improve sleep quality and executive functions through the regulation of gut microbiota.

## Graphical abstract

Gut microbiota can modulate large‐scale inter‐ and intranetwork functional connectivity (especially the executive control system) in young adulthood. Some functional network connectivity may act as mediators of the effects of gut microbiota on sleep and executive functions.

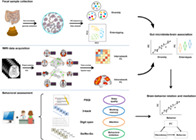

## 1 INTRODUCTION

The term microbiota–gut–brain axis refers to the bidirectional conduit that communicates the brain and the gut microbiota (Cryan et al., 2019). Gut–brain interactions have received increasing attention in recent years, such that numerous findings suggest a fundamental influence of the gut microbiota on brain development and function (Dinan & Cryan, 2017). It is generally assumed that the gut microbiota can affect and be reciprocally affected by many behavioral factors, including social function, cognition, emotion, stress, and food intake (Johnson & Foster, 2018; Sherwin, Bordenstein, Quinn, Dinan, & Cryan, 2019; Vuong, Yano, Fung, & Hsiao, 2017). Alterations in gut microbiota have been associated with a wide range of brain disorders, such as Alzheimer's disease, depression, and autism (Cryan, O'Riordan, Sandhu, Peterson, & Dinan, 2020; Van Ameringen et al., 2019).

Mounting preclinical evidence has implicated that there are many pathways of potential communication between the gut microbiota and the brain, such as autonomic nervous system (Fulling, Dinan, & Cryan, 2019), enteric nervous system (Furness, 2012), immune system and neuroimmunity (Fung, Olson, & Hsiao, 2017), enteroendocrine signaling, neurotransmitters (Agus, Planchais, & Sokol, 2018; Dodd et al., 2017; Strandwitz et al., 2019), branched chain amino acids, short‐chain fatty acids (Dalile, Van Oudenhove, Vervliet, & Verbeke, 2019), spinal mechanisms, and hypothalamic–pituitary–adrenal axis. However, the exact mechanism of such interaction in humans is still largely unclear. A full explanation of this important issue may provide scientific basis for the potential usefulness of the microbiota–gut–brain axis as biological markers for accurate diagnosis and effective treatment of brain disorders.

Advances in neuroimaging and microbiome sequencing techniques have made it increasingly feasible to probe the interactions between the brain, gut, and microbiome in healthy and clinical populations (De Santis, Moratal, & Canals, 2019; Liu, Peng, Zhang, Wang, & Luo, 2019; Mayer et al., 2019). For example, using functional magnetic resonance imaging (fMRI), Curtis et al. (2019) demonstrated that insular functional connectivity was associated with microbiome diversity and structure in healthy young participants. A previous study in infants showed that gut microbial diversity was linked to functional connectivity of multiple brain regions (Gao et al., 2019). Tillisch et al. (2017) found that the gut microbial profiles were related to task‐based brain activity, gray matter metric as well as white matter fiber density in healthy women. Moreover, some longitudinal studies have demonstrated significant influence of probiotic administration on resting‐state functional connectivity and task‐related brain activity in healthy young subjects (Bagga et al., 2018, 2019; Tillisch et al., 2013). For patients with irritable bowel syndrome (IBS), probiotic administration reduced depression and increased quality of life, and these improvements were accompanied by changes in brain activation patterns (Pinto‐Sanchez et al., 2017). In addition, Labus et al. found links of gut microbes with resting‐state functional connectivity and regional brain volumes in patients with IBS (Labus et al., 2017; Labus et al., 2019). A recent study revealed an inner relationship between gut microbiota alterations, systemic inflammation, default mode network (DMN) dysfunction and cognitive impairment in patients with end‐stage renal disease (Y. F. Wang et al., 2019). There is also evidence for the impact of obesity on potential interactions among gut microbiota composition, brain microstructure, and cognition (Fernandez‐Real et al., 2015).

It is well established that the brain is a complex system consisting of multiple functional networks subserving different functions (Damoiseaux et al., 2006; De Luca, Beckmann, De Stefano, Matthews, & Smith, 2006; Power et al., 2011). Each functional network is composed of several brain regions with similar patterns of blood‐oxygen‐level‐dependent (BOLD) signal change over the course of resting‐state fMRI, whereas different networks show distinct patterns. Independent component analysis (ICA) of resting‐state fMRI data represents a useful data‐driven method that can identify and extract these different functional networks, and further investigate inter‐ and intranetwork functional connectivity (Calhoun, Adali, Pearlson, & Pekar, 2001; van de Ven, Formisano, Prvulovic, Roeder, & Linden, 2004). This approach has been broadly applied to the domain of neuroscience and has enjoyed significant success in unraveling the large‐scale functional organization in normal and abnormal brains (Barkhof, Haller, & Rombouts, 2014; Buckner & Vincent, 2007; Fox & Raichle, 2007). However, little is known, so far, about the relationship of the gut microbiome with functional connectivity between and within large‐scale functional networks.

In the current work, we collected fecal samples from a large and homogeneous sample of healthy young adults and used 16S rRNA gene amplicon sequencing to assess gut microbial diversity and enterotypes (Arumugam et al., 2011; Claesson, Clooney, & O'Toole, 2017). Large‐scale inter‐ and intranetwork functional connectivity was computed using a combination of resting‐state fMRI data and ICA approach. In addition, Pittsburgh Sleep Quality Index (PSQI) and a set of neuropsychological experimental paradigms (i.e., 3‐back, digit span, and Go/No‐Go tasks) were employed to assess sleep quality (Buysse, Reynolds 3rd, Monk, Berman, & Kupfer, 1989) and core executive functions including working memory (Owen, McMillan, Laird, & Bullmore, 2005), attention (Groth‐Marnat & Baker, 2003), and behavioral inhibition (Kaufman, Ross, Stein, & Garavan, 2003). The focus was set on these behaviors due to their close associations with the gut microbiota (Arnoriaga‐Rodriguez et al., 2020; Cenit, Nuevo, Codoner‐Franch, Dinan, & Sanz, 2017; Grosicki, Riemann, Flatt, Valentino, & Lustgarten, 2020).

**FIGURE 1:**
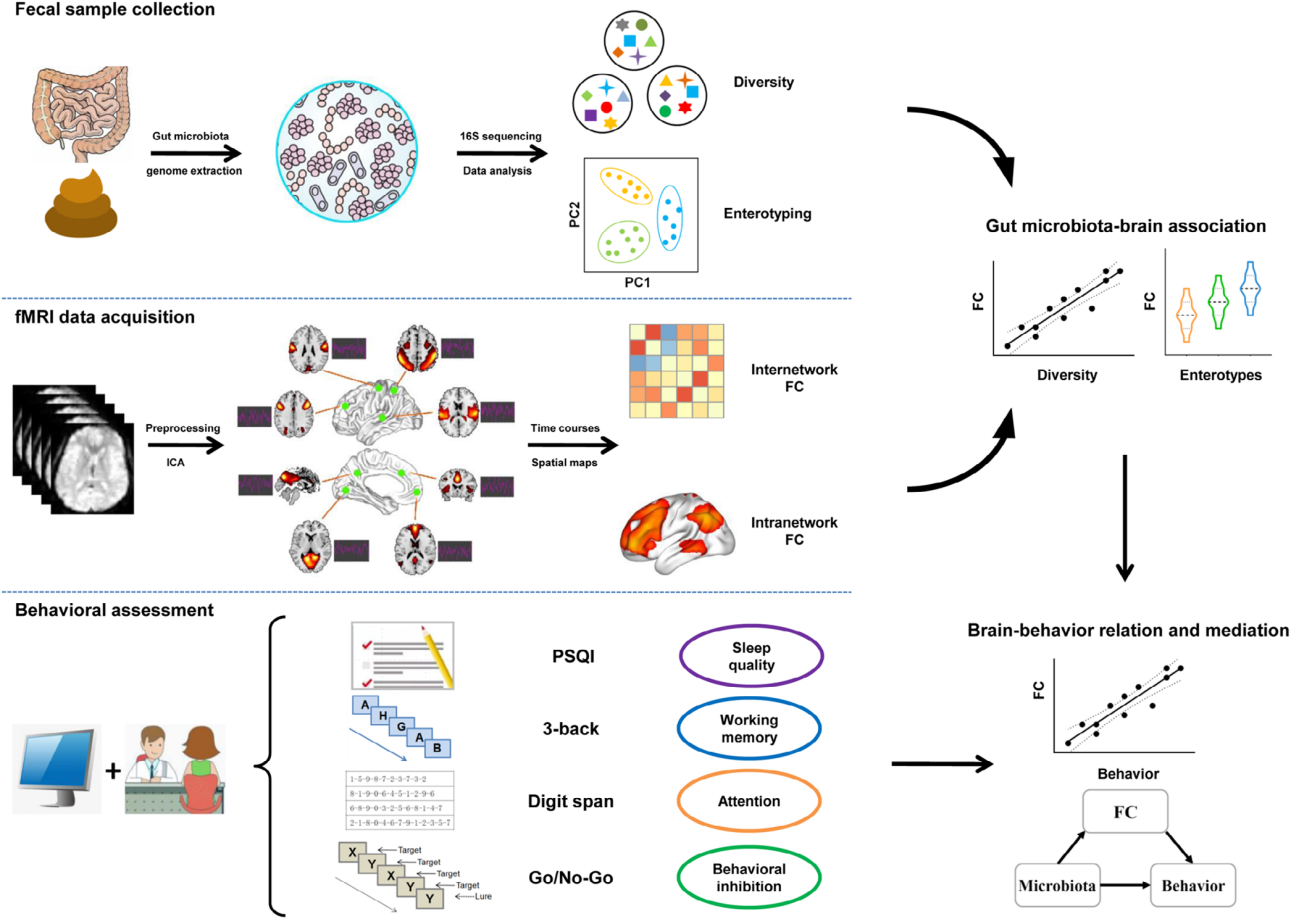
Flowchart of the study design. FC, functional connectivity; fMRI, functional magnetic resonance imaging; ICA, independent component analysis; PSQI, Pittsburgh Sleep Quality Index

By a combined analysis of these data, our objectives in this investigation were three‐fold. First, we attempted to assess the relationship between the gut microbiota and the brain by testing the associations of inter‐ and intranetwork functional connectivity with gut microbial diversity and enterotypes. Second, we aimed to investigate the potential associations of gut microbiota‐linked functional connectivity with sleep quality and executive functions. Finally, we sought to establish the meditative role of these identified functional connectivity markers in accounting for the relations between gut microbiota and behaviors. A systematic flowchart of the study design is shown in Figure 1. We hypothesized that the gut microbiota would be associated with functional network connectivity, which would mediate the relations between gut microbiota and behaviors.

## 2 MATERIALS AND METHODS

### 2.1 Participants

A total of 157 healthy young adults were recruited by advertisement. All participants met the inclusion criteria of Chinese Han, right handedness, and within a restricted age range of 18–30 years, which corresponds to a period after the completion of major neurodevelopment and before the onset of neurodegenerative changes. Exclusion criteria included neuropsychiatric or severe somatic disorder, a history of alcohol or drug abuse, regular smokers (i.e., total number of cigarettes >20), current medication (e.g., antibiotics or sedative hypnotics) within a month, pregnancy, MRI contraindications, and a family history of psychiatric illness among first‐degree relatives. The MINI‐International Neuropsychiatric Interview (M.I.N.I.) and Alcohol Use Disorders Identification Test (AUDIT) were used in the process of excluding participants. The participants' dietary habit information was collected using the Dietary Nutrition and Health Questionnaires (DNHQ), which is a self‐report questionnaire including 50 items. The total scores range from 50 to 200 points, with lower score reflecting better dietary habits. The participants' physical exercise was evaluated using the International Physical Activity Questionnaires (IPAQ) (short self‐administrated format), which is a 4‐item self‐reported measure of physical activity in last 7 days. This study was approved by the ethics committee of The First Affiliated Hospital of Anhui Medical University. Written informed consent was obtained from all participants after they had been given a complete description of the study. Detailed data of the participants are listed in Table 1.

**TABLE 1:** Demographic, gut microbial, and behavioral characteristics of the participants

Characteristics	Mean ± *SD*	Range
Gender (female/male)	77/80	—
Age (years)	22.32 ± 2.42	18–28
Education (years)	15.78 ± 1.92	12–20
BMI (kg/m^2^)	21.44 ± 3.20	15.42–36.99
DNHQ	101.94 ± 10.43	77–127
IPAQ	2,128.36 ± 1,553.39	66–10,444
*Alpha diversity*		
Sobs	264.52 ± 60.21	122–398
Ace	307.51 ± 67.12	162.93–471.62
Chao	310.23 ± 69.24	158.43–477.07
Shannon	3.06 ± 0.52	1.61–3.97
Simpson	0.13 ± 0.09	0.03–0.51
Enterotypes (P/R/B)	51/37/69	
Total score of PSQI	3.66 ± 2.00	0–11
*3‐back task performance*		
Accuracy	0.72 ± 0.16	0.15–0.98
Reaction time (ms)	768.87 ± 175.24	230.23–1,179.93
*Digit span task performance*		
Digit span forward	8.49 ± 1.29	5–13
Digit span backward	6.57 ± 1.54	3–10
*Go/No‐Go task performance*		
Acc_No‐Go	0.59 ± 0.19	0.05–1.00
Acc_Go	0.95 ± 0.10	0.47–1.00
RT_Go (ms)	432.83 ± 69.57	256.73–591.64
FD (mm)	0.12 ± 0.05	0.04–0.40

Abbreviations: Acc_No‐Go, accuracy in “No‐Go” conditions; Acc_Go, accuracy in “Go” conditions; B, bacteroides; BMI, body mass index; DNHQ, Dietary Nutrition and Health Questionnaires; FD, frame‐wise displacement; IPAQ, International Physical Activity Questionnaires; P, prevotella; PSQI, Pittsburgh Sleep Quality Index; R, ruminococcaceae; RT_Go, mean reaction time of correct responses in “Go” conditions; *SD*, standard deviation.

### 2.2 Sleep quality assessment

Self‐reported sleep habits over a 1‐month time span were assessed with the PSQI (Buysse, Reynolds 3rd, Monk, Berman, & Kupfer, 1989). This index is a self‐rated questionnaire and consists of 17 items with the majority using a 4‐point Likert‐type scale. The PSQI provides a comprehensive assessment of sleep quality by generating seven components: subjective sleep quality, sleep latency, sleep duration, habitual sleep efficiency, sleep disturbances, use of sleep medication, and daytime dysfunction. By summing component scores, a total score of PSQI is generated ranging from 0 to 21, with a lower score indicating greater sleep quality and a score of higher than five signifying the presence of significant sleep disturbance (Buysse et al., 1989).

### 2.3 Cognition assessment

The letter 3‐back task was conducted on a computer to assess working memory (Owen, McMillan, Laird, & Bullmore, 2005) using E‐Prime 2.0 (http://www.pstnet.com/eprime.cfm). During the task, each participant viewed a series of letters that were presented sequentially and the presentation time of each letter stimulus was 200 ms with an inter‐stimulus interval of 1,800 ms. Participants were instructed to press a button on the right with their middle fingers if the letter that appeared on the screen was identical to the one presented three letters earlier, and otherwise to press a button on the left with their index fingers. The task consisted of 60 trials. Before the formal test, participants were verbally instructed and had a practice test to ensure that they understood the task. The accuracy and mean reaction time of correct responses were used as the indices of working memory performance.

We also adopted digit span tasks to evaluate attention (Groth‐Marnat & Baker, 2003). All participants completed a digit span forward task followed by a digit span backward task. The former begins with a series of two digits orally presented to each participant continuing to a maximum of 13 digits. Participants were asked to verbally repeat the digits. There were two trials per digit series. All participants began with the first digit series (i.e., two digits), if repeated correctly, the participant continued to the next one, otherwise performed the second trial at the same digit series. The task was discontinued when the participant failed in the second trial. The span is defined as the maximum number of digits repeated by the participant. The digit span backward task followed the same procedure, except that participants verbally repeated the digits in reverse order.

The Go/No‐Go task was performed on a computer to assess the ability of behavioral inhibition (Kaufman, Ross, Stein, & Garavan, 2003) using E‐Prime 2.0. During the task, the letter X or Y was presented at a frequency of 1 Hz on the screen. In “Go” conditions, the current letter is different from the previous one and participants should respond quickly by pressing the button within 900 ms. In “No‐Go” conditions (10% of all trials), the current letter is the same as the previous one and participants cannot press the button; if one presses the button, it would be counted as an error. The Go/No‐Go task consisted of a practice test and a formal test. There were 20 trials (15 “Go” trials and 5 “No‐Go” trials) in the practice test. If a participant responds correctly in three “No‐Go” trials, he or she can shift to the formal test; otherwise, the participant needs to restart the practice test. The formal test was divided into two groups with 210 trials in each group and 30 s break between the two groups. It took about 12 min for the Go/No‐Go task. The accuracy in “No‐Go” conditions (Acc_No‐Go) as well as the accuracy and mean reaction time of correct responses in “Go” conditions (Acc_Go and RT_Go) were used as the indices of task performance.

### 2.4 Fecal samples collection and gut microbiota analysis

Fecal samples were collected in sterilized tubes and stored immediately in a −80°C freezer within 1 day before or after MRI examination. Microbial genome DNA was extracted from the fecal samples using a QIAamp DNA Stool Mini Kit (Qiagen Inc., Hilden, Germany). To construct the Polymerase Chain Reaction (PCR)‐based 16S rDNA amplicon library for sequencing, PCR enrichment of the V4 hypervariable region of 16S rDNA was performed with the forward primer 515F (5′‐GTGCCAGCMGCCGCGGTAA‐3′) and reverse primer 806R (5′‐GGACTACHVGGGTWTCTAAT‐3′). The qualified amplicon mixture was then sequenced on the MiSeq platform with the PE250 sequencing strategy. Before the 16S rDNA data analysis, raw reads were filtered to remove adaptors and low‐quality and ambiguous bases, and then paired‐end reads were added to tags by the Fast Length Adjustment of Short reads program (FLASH, v1.2.11; Magoc & Salzberg, 2011). The tags were clustered into operational taxonomic units (OTUs) with a cutoff value of 97% using UPARSE software (v9.1.13) (Edgar, 2013) and chimera sequences were compared with the Gold database using UCHIME (v4.2.40) (Edgar, Haas, Clemente, Quince, & Knight, 2011) to detect. Then, the representative sequence from each OTU cluster was obtained. These OTU representative sequences were taxonomically classified using Ribosomal Database Project (RDP) Classifier (v.2.2) (Q. Wang, Garrity, Tiedje, & Cole, 2007) with a minimum confidence threshold of 0.8, and the training database was the Greengene Database (v201305) (DeSantis et al., 2006). The USEARCH_global (Edgar, 2010) was used to compare all tags back to OTU to get the OTU abundance statistics table of each sample.

Alpha diversity was assessed using the species richness indices (Sobs, Ace and Chao) and species diversity indices (Shannon and Simpson that reflect both species richness and species evenness; Keylock, 2005; Faith, 1992), which were calculated by MOTHUR (v1.31.2) (Schloss et al., 2009) and QIIME (v1.8.0) (Caporaso et al., 2010) at the OTU level. Of note, there were significant correlations between these alpha diversity indices (Table S1). Sample enterotyping was performed based on OTU‐derived genus abundance matrix as described in the original publication (Arumugam et al., 2011). Specifically, samples were clustered using Jensen–Shannon distance and partitioning around medoid (PAM) clustering. Calinski–Harabasz (CH) index was used to assess optimal number of clusters. The silhouette validation technique was utilized to assess the robustness of clusters.

### 2.5 MRI data acquisition

MRI scans were obtained using a 3.0‐Tesla MR system (Discovery MR750w, General Electric, Milwaukee, WI) with a 24‐channel head coil. Earplugs were used to reduce scanner noise, and tight but comfortable foam padding was used to minimize head motion. High‐resolution 3D T1‐weighted structural images were acquired by employing a brain volume (BRAVO) sequence with the following parameters: repetition time (TR) = 8.5 ms; echo time (TE) = 3.2 ms; inversion time (TI) = 450 ms; flip angle (FA) = 12°; field of view (FOV) = 256 mm × 256 mm; matrix size = 256 × 256; slice thickness = 1 mm, no gap; 188 sagittal slices; and acquisition time = 296 s. Resting‐state BOLD fMRI data were acquired using a gradient‐echo single‐shot echo planar imaging (GRE‐SS‐EPI) sequence with the following parameters: TR = 2,000 ms; TE = 30 ms; FA = 90°; FOV = 220 mm × 220 mm; matrix size = 64 × 64; slice thickness = 3 mm, slice gap = 1 mm; 35 interleaved axial slices; 185 volumes; and acquisition time = 370 s. Before the scanning, all subjects were instructed to keep their eyes closed, relax, move as little as possible, think of nothing in particular, and not fall asleep during the scans. During and after the scanning, we asked subjects whether they had fallen asleep to confirm that none of them had done so. All MR images were visually inspected to ensure that only images without visible artifacts were included in subsequent analyses.

### 2.6 fMRI data preprocessing

Resting‐state BOLD data were preprocessed using Statistical Parametric Mapping software (SPM12, http://www.fil.ion.ucl.ac.uk/spm) and Data Processing & Analysis for Brain Imaging (DPABI, http://rfmri.org/dpabi; Yan, Wang, Zuo, & Zang, 2016). The first 10 volumes for each participant were discarded to allow the signal to reach equilibrium and the participants to adapt to the scanning noise. The remaining volumes were corrected for the acquisition time delay between slices. Then, realignment was performed to correct the motion between time points. Head motion parameters were computed by estimating the translation in each direction and the angular rotation on each axis for each volume. All participants' BOLD data were within the defined motion thresholds (i.e., translational or rotational motion parameters less than 2 mm or 2°). We also calculated frame‐wise displacement (FD), which indexes the volume‐to‐volume changes in head position. In the normalization step, individual structural images were firstly co‐registered with the mean functional image; then the transformed structural images were segmented and normalized to the Montreal Neurological Institute (MNI) space using a high‐level nonlinear warping algorithm, that is, the diffeomorphic anatomical registration through the exponentiated Lie algebra (DARTEL) technique (Ashburner, 2007). Finally, each filtered functional volume was spatially normalized to MNI space using the deformation parameters estimated during the above step and resampled into a 3‐mm cubic voxel. After spatial normalization, all data sets were smoothed with a Gaussian kernel of 6 × 6 × 6 mm^3^ full‐width at half maximum.

### 2.7 Independent component analysis

ICA was conducted to parcellate the preprocessed fMRI data with the GIFT toolbox (mialab.mrn.org/software/gift/) and the number of independent components (*N* = 26) was estimated automatically by the software using the minimum description length criteria. Spatial ICA decomposes the participant data into linear mixtures of spatially independent components that exhibit a unique time course profile. This was achieved by using two data reduction steps. First, principal component analysis was applied to reduce the subject‐specific data into 39 principle components. Next, reduced data of all subjects were concatenated across time and decomposed into 26 independent components using the infomax algorithm. To ensure estimation stability, the infomax algorithm was repeated 20 times in ICASSO (http://research.ics.tkk.fi/ica/icasso/), and the most central run was selected and analyzed further. Finally, participant specific spatial maps and time courses were obtained using the GICA back reconstruction approach.

**FIGURE 2:**
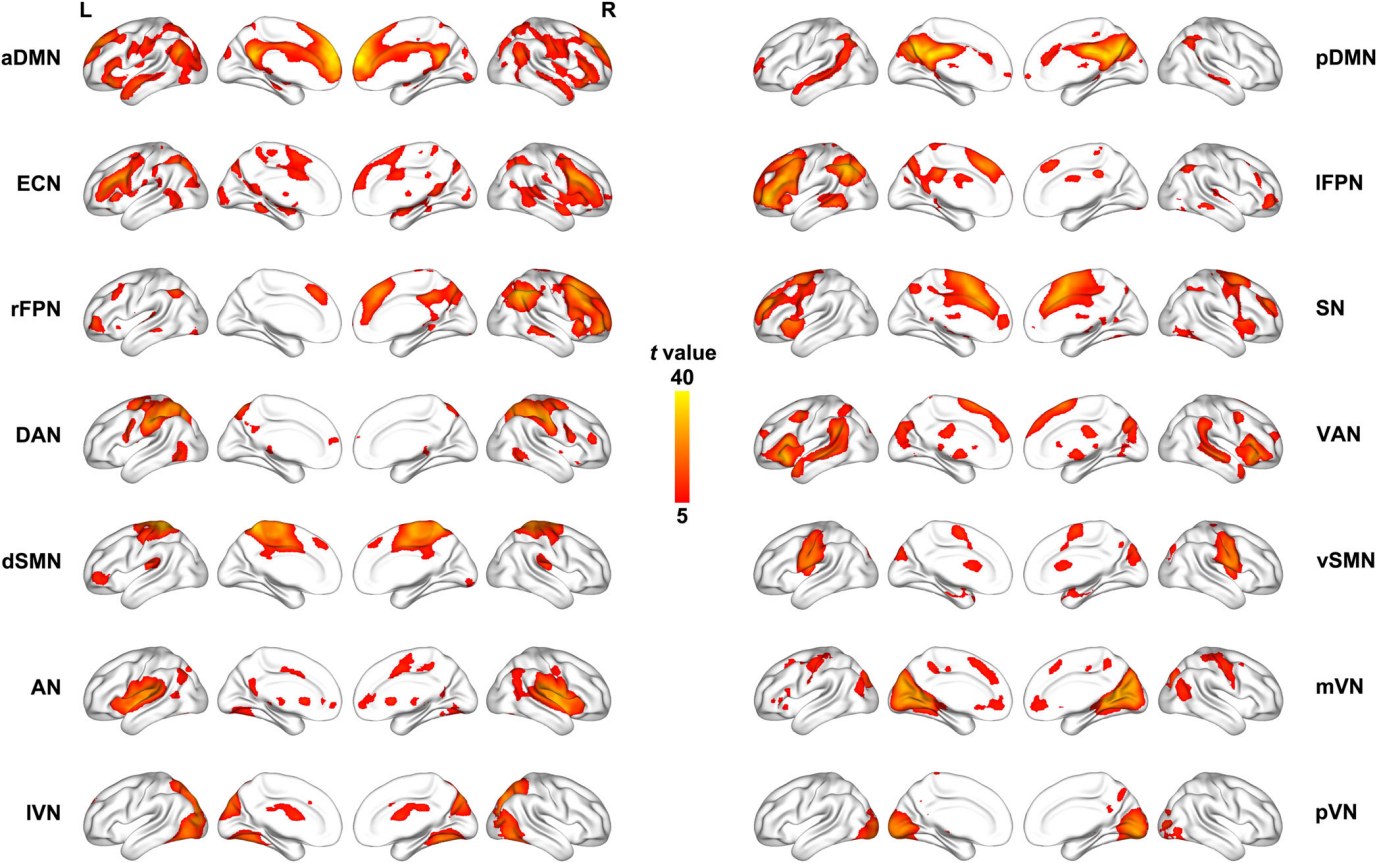
Spatial maps of 14 selected functional networks. aDMN, anterior default mode network; AN, auditory network; dSMN, dorsal sensorimotor network; DAN, dorsal attention network; ECN, executive control network; L, left; lFPN, left frontoparietal network; lVN, lateral visual network; mVN, medial visual network; pDMN, posterior default mode network; pVN, posterior visual network; R, right; rFPN, right frontoparietal network; SN, salience network; VAN, ventral attention network; vSMN, ventral sensorimotor network

We identified as functional networks several independent components that had peak activations in gray matter, showed low spatial overlap with known vascular, ventricular, motion, and susceptibility artifacts, and exhibited primarily low frequency power. This selection procedure resulted in 14 functional networks out of the 26 independent components obtained (Figure 2): anterior and posterior default mode networks (aDMN and pDMN), executive control network (ECN), left and right frontoparietal networks (lFPN and rFPN), salience network (SN), dorsal and ventral attention networks (DAN and VAN), dorsal and ventral sensorimotor networks (dSMN and vSMN), auditory network (AN), medial, lateral, and posterior visual networks (mVN, lVN, and pVN).

Before internetwork functional connectivity calculation, the following additional postprocessing steps were performed on the time courses of selected functional networks: (1) detrending linear, quadratic, and cubic trends; (2) despiking detected outliers; and (3) low‐pass filtering with a cut‐off frequency of 0.15 Hz. Then, internetwork functional connectivity was estimated as the Pearson correlation coefficients between pairs of time courses of the functional networks, resulting in a symmetric 14 × 14 correlation matrix for each subject. Finally, correlations were transformed to *z*‐scores using Fisher's transformation to improve the normality. Intranetwork connectivity was examined via the spatial maps, indexing the contribution of the time course to each voxel comprising a given component.

### 2.8 Statistical analysis

The statistical descriptive analyses of demographic, gut microbial, and behavioral data were conducted using the SPSS 23.0 software package (SPSS, Chicago, IL). We adopted a multi‐stage approach to analyze the data of gut microbiota (alpha diversity and enterotypes), neuroimaging (inter‐ and intranetwork functional connectivity), and behaviors (sleep quality and executive functions). First, we tested for the gut microbiota‐brain associations by performing partial correlation analyses between alpha diversity and functional connectivity with age, sex, and FD as nuisance covariates, followed by performance of group comparisons in functional connectivity across enterotypes using one‐way analysis of variance (ANOVA). For internetwork functional analysis, multiple comparisons were corrected by false discovery rate (FDR) with a corrected significance level of *p* <.05. For intranetwork functional analysis, all participants' spatial maps for each functional network were initially entered into a random‐effect one‐sample *t*‐test. Brain regions were considered to be within each network if they met a height threshold of *p* <.05 corrected for multiple comparisons using a family‐wise error (FWE) and an extent threshold of 100 voxels. Next, we performed the above‐described analyses (correlations followed by group comparisons) in a voxel‐wise manner within each network. Multiple comparisons were corrected using the cluster‐level FWE method, resulting in a cluster defining threshold of *p* = 0.001 and a corrected cluster significance of *p* <.05. Second, for inter‐ and intranetwork functional connectivity showing correlations with alpha diversity or differences across enterotypes, we further examined their associations with behavioral variables using partial correlations adjusting for age, sex and FD. Finally, to further test whether the relationship between gut microbiota and behaviors was mediated by functional connectivity, mediation analysis was performed using the PROCESS macro (http://www.processmacro.org/). In the mediation models, all paths were reported as unstandardized ordinary least squares regression coefficients, namely, total effect of *X* on *Y* (*c*) = indirect effect of *X* on *Y* through *M* (*a* × *b*) + direct effect of *X* on *Y* (*c*′). The significance analysis was based on 10,000 bootstrap realizations and a significant indirect effect is indicated when the bootstrap 95% confidence interval (CI) does not include zero. Here, only variables that demonstrated a significant association with others were considered independent (gut microbiota), dependent (behaviors), or mediating (functional connectivity) variables in the mediation analysis. Age, sex, and FD were considered nuisance variables.

### 2.9 Sensitivity analysis

To test the possible effect of body mass index (BMI) on our results, we included BMI as an additional nuisance covariate in the analyses of gut microbiota‐brain associations. To examine the possibility that our main results were not influenced by dietary habit and physical exercise, we also included DNHQ and IPAQ scores as additional nuisance covariates in the analyses.

## 3 RESULTS

### 3.1 Associations between microbial diversity, functional connectivity, and behaviors

**FIGURE 3:**
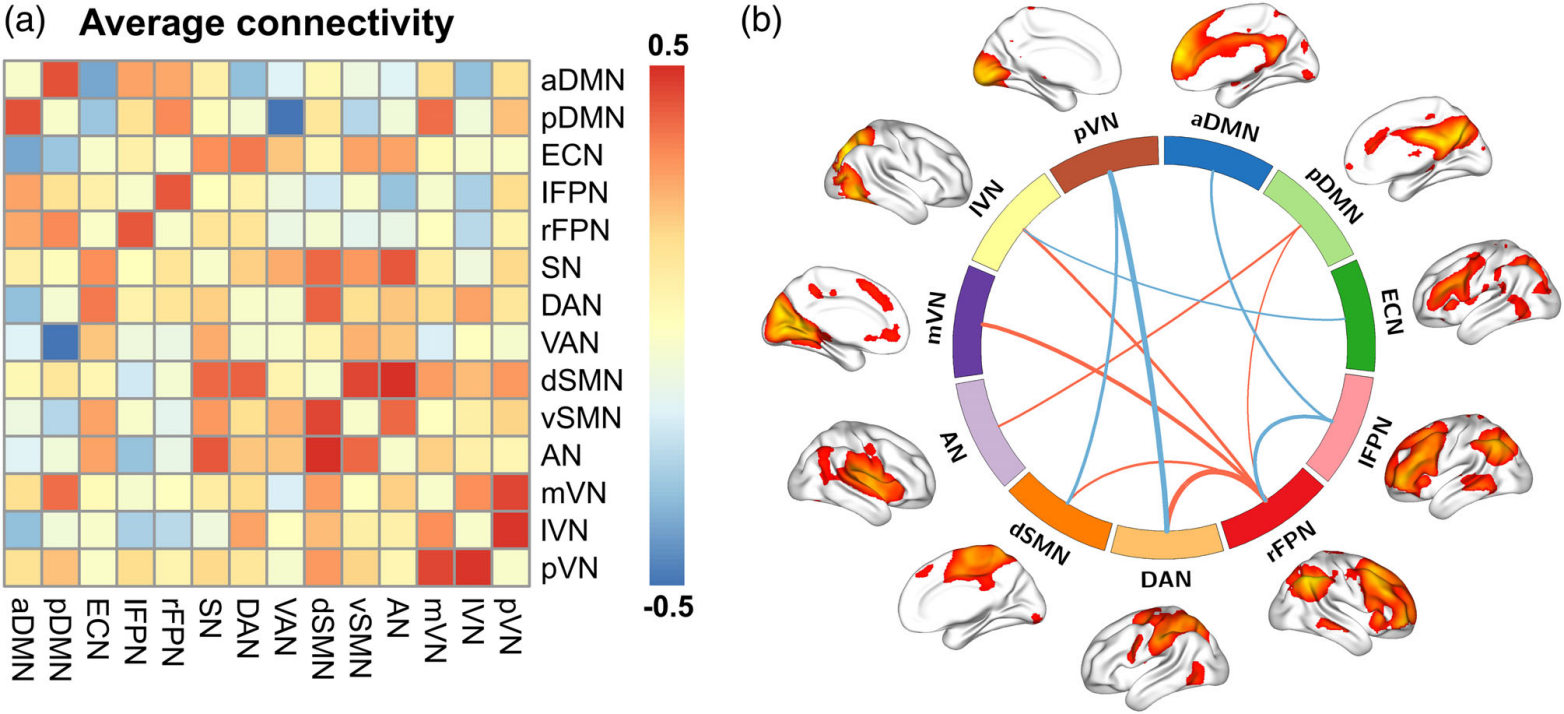
(a) Internetwork functional connectivity matrix. Pairwise correlations between functional networks were averaged across subjects. Hot colors represent positive functional connectivity and cool colors represent negative functional connectivity. (b) Associations between Simpson index and internetwork functional connectivity. Line thickness denotes magnitude of the correlation coefficients between Simpson index and internetwork functional connectivity, with hot and cool colors representing positive and negative correlations, respectively. aDMN, anterior default mode network; AN, auditory network; DAN, dorsal attention network; dSMN, dorsal sensorimotor network; ECN, executive control network; lFPN, left frontoparietal network; lVN, lateral visual network; mVN, medial visual network; pDMN, posterior default mode network; pVN, posterior visual network; rFPN, right frontoparietal network; SN, salience network; VAN, ventral attention network; vSMN, ventral sensorimotor network

Pairwise correlation patterns between functional networks are illustrated in Figure 3a. Both positive and negative internetwork functional connectivity were observed. Correlation analyses revealed significant correlations between Simpson index and internetwork functional connectivity (*p* <.05, FDR corrected; Figure 3b). Specifically, Simpson index was positively correlated with functional connectivity between pDMN and rFPN (*t* = 2.47, *p* = .0145), between pDMN and AN (*t* = 2.68, *p* = .0082), between rPFN and DAN (*t* = 3.50, *p* = .0006), between rPFN and dSMN (*t* = 2.69, *p* = .0081), between rPFN and mVN (*t* = 3.08, *p* = .0024), and between rPFN and lVN (*t* = 2.90, *p* = .0044), as well as negatively correlated with connectivity between aDMN and lFPN (*t* = −2.57, *p* = .0111), between ECN and lVN (*t* = −2.46, *p* = .0150), between lFPN and rFPN (*t* = −2.75, *p* = .0067), between DAN and pVN (*t* = −2.98, *p* = .0033), and between dSMN and pVN (*t* = −2.65, *p* = .0089). However, there were no significant correlations between internetwork functional connectivity and other microbial diversity indices.

**FIGURE 4:**
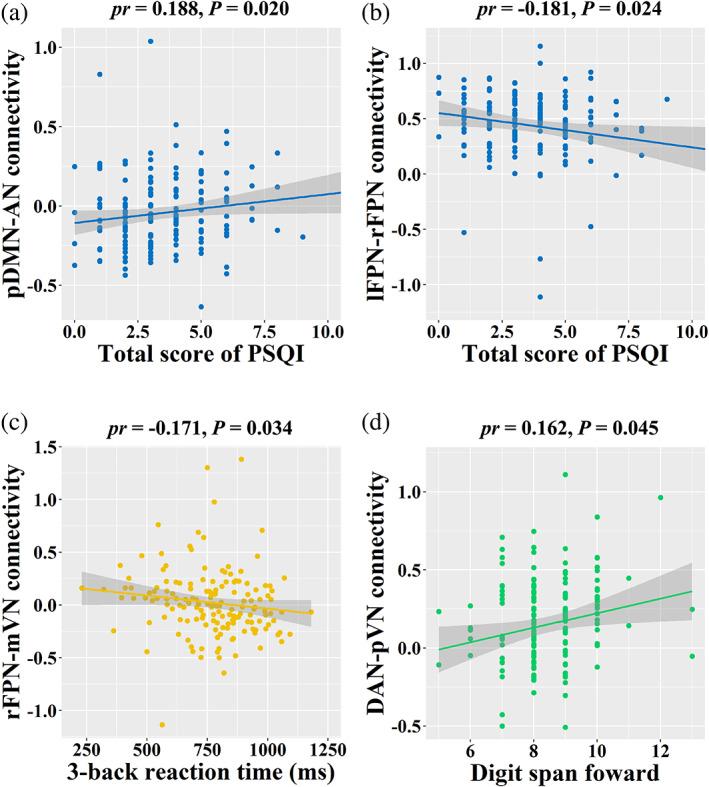
Correlations between internetwork functional connectivity and behaviors. AN, auditory network; DAN, dorsal attention network; lFPN, left frontoparietal network; mVN, medial visual network; pDMN, posterior default mode network; PSQI, Pittsburgh Sleep Quality Index; pVN, posterior visual network; rFPN, right frontoparietal network

**FIGURE 5:**
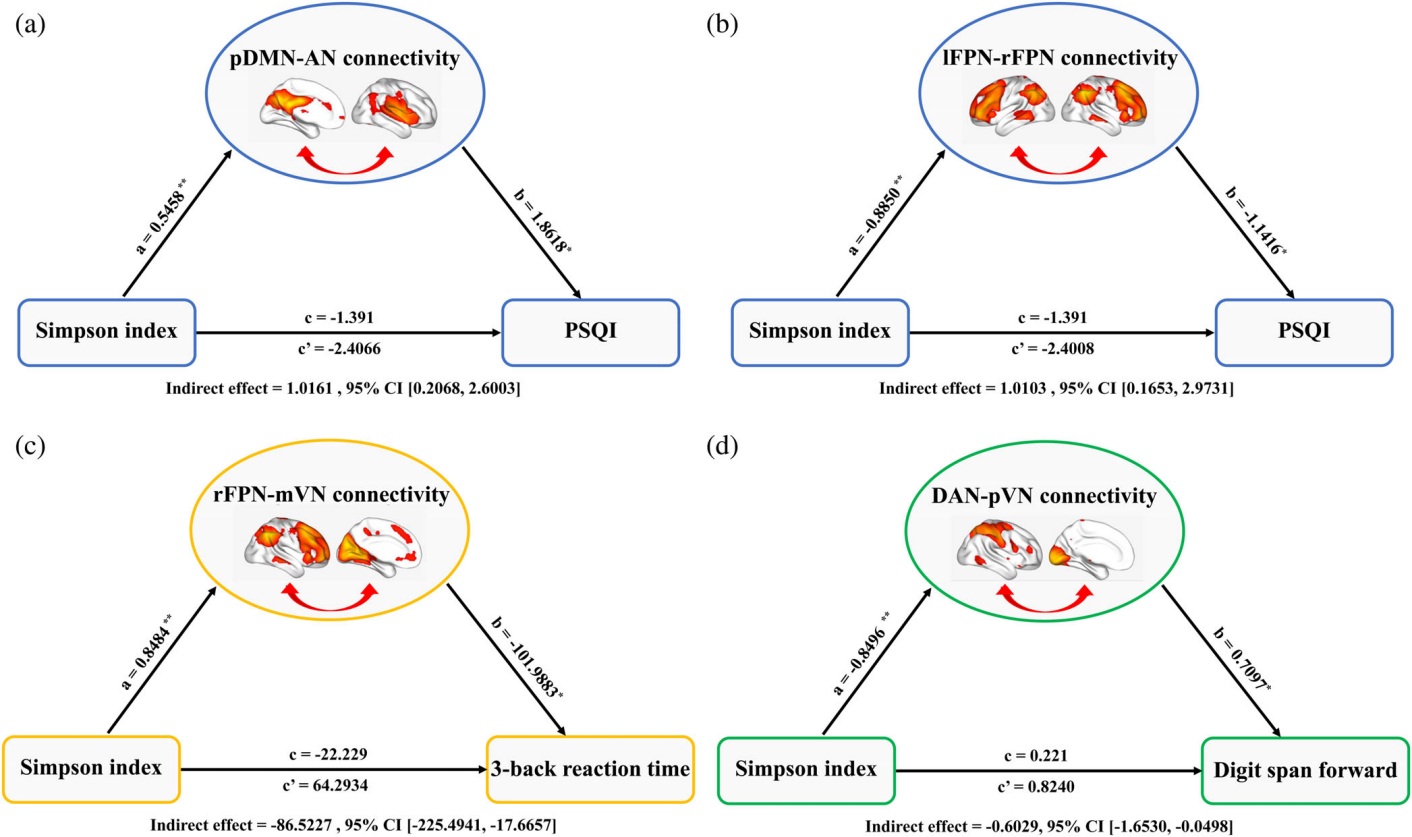
Gut microbial diversity‐internetwork functional connectivity‐behaviors associations. (a) and (b) The mediation analyses between Simpson index (X) and total score of PSQI (Y), with pDMN‐AN and lFPN‐rFPN connectivity as the mediators (M). (c) The mediation analysis between Simpson index (X) and 3‐back reaction time (Y), with rFPN‐mVN connectivity as the mediator. (d) The mediation analysis between Simpson index (X) and digit span forward (Y), with DAN‐pVN connectivity as the mediator. Path coefficients with *p* values (*p <.05 and **p <.01, respectively). AN, auditory network; DAN, dorsal attention network; lFPN, left frontoparietal network; mVN, medial visual network; pDMN, posterior default mode network; PSQI, Pittsburgh Sleep Quality Index; pVN, posterior visual network; rFPN, right frontoparietal network

With regard to sleep quality, total score of PSQI was found to be positively correlated with pDMN‐AN connectivity (*pr* = .188, *p* = .020; Figure 4a) and negatively correlated with lFPN‐rFPN connectivity (*pr* = −.181, *p* = .024; Figure 4b). Further mediation analyses revealed that pDMN‐AN (indirect effect = 1.0161, standard error [*SE*] = 0.5654, 95% CI: 0.2068, 2.6003) and lFPN‐rFPN (indirect effect = 1.0103, *SE* = 0.6048, 95% CI: 0.1653, 2.9731) connectivity mediated the relationship between Simpson index and total score of PSQI (Figure 5a,b). In term of working memory, there was a significant negative correlation between rFPN‐mVN connectivity and 3‐back reaction time (*pr* = −.171, *p* = .034) (Figure 4c). Likewise, rFPN‐mVN connectivity mediated the relationship between Simpson index and 3‐back reaction time (indirect effect = −86.5227, *SE* = 49.2462, 95% CI: −225.4941, −17.6657; Figure 5c). With respect to attention, we found a significant positive correlation between DAN‐pVN connectivity and digit span forward (*pr* = .162, *p* = .045; Figure 4d). Further mediation analysis showed that DAN‐pVN connectivity mediated the relationship between Simpson index and digit span forward (indirect effect = −0.6029, *SE* = 0.3796, 95% CI: −1.6530, −0.0498; Figure 5d).

**FIGURE 6:**
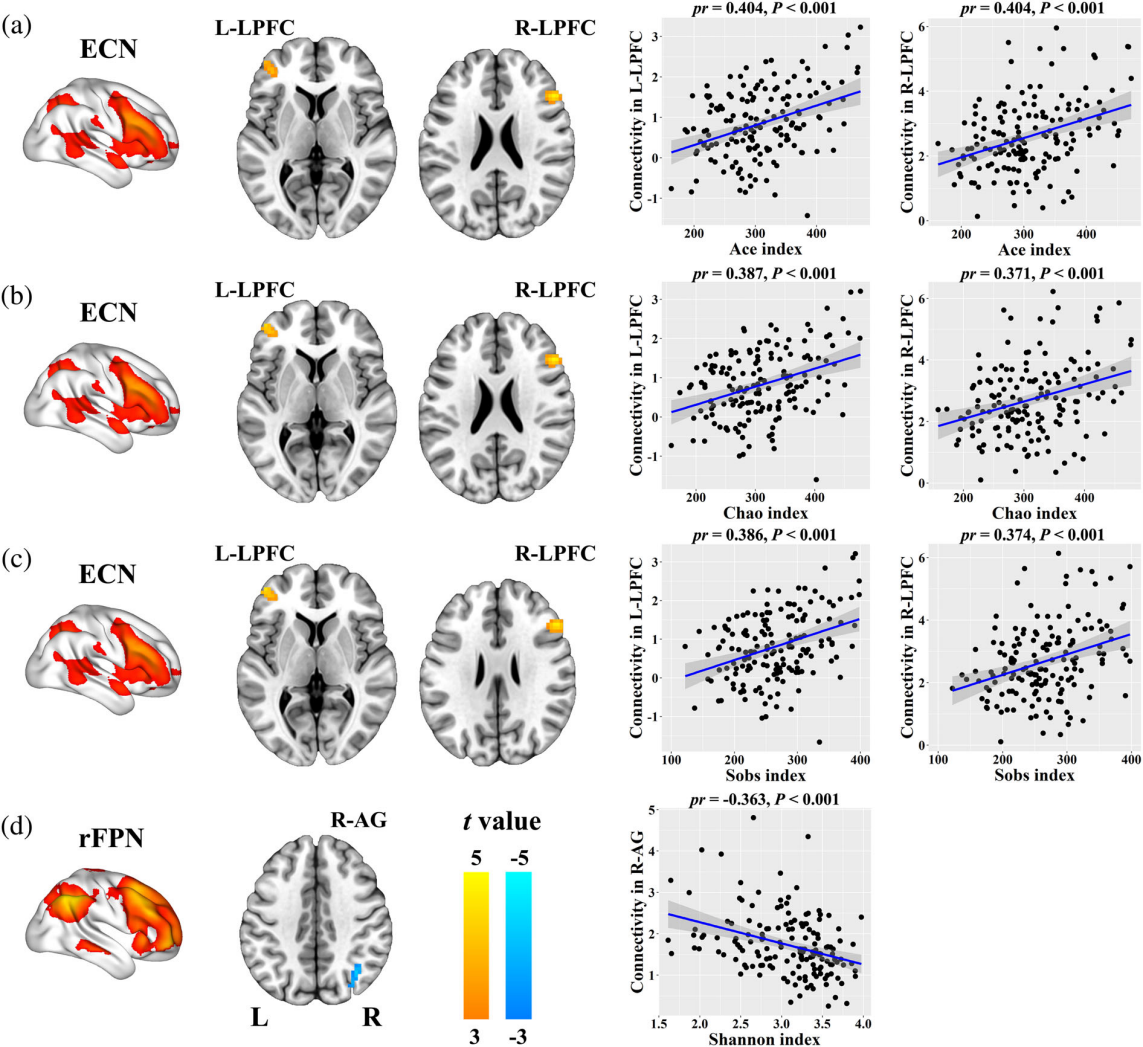
Correlations between gut microbial diversity and intranetwork functional connectivity. AG, angular gyrus; ECN, executive control network; L, left; LPFC, lateral prefrontal cortex; R, right; rFPN, right frontoparietal network

Voxel‐wise intranetwork functional connectivity analyses demonstrated significant positive correlations between Ace index and intranetwork connectivity in the bilateral lateral prefrontal cortex (LPFC) (left: cluster size = 47 voxels, peak MNI coordinate *x*/*y*/*z* = −45/48/0, peak *t* = 5.02; right: cluster size = 45 voxels, peak MNI coordinate *x*/*y*/*z* = 51/27/27, peak *t* = 5.28) of the ECN (Figure 6a), between Chao index and intranetwork connectivity in the bilateral LPFC (left: cluster size = 37 voxels, peak MNI coordinate *x*/*y*/*z* = −45/48/0, peak *t* = 4.70; right: cluster size = 34 voxels, peak MNI coordinate *x*/*y*/*z* = 54/27/27, peak *t* = 4.85) of the ECN (Figure 6b) and between Sobs index and intranetwork connectivity in the bilateral LPFC (left: cluster size = 35 voxels, peak MNI coordinate *x*/*y*/*z* = −45/48/0, peak *t* = 4.85; right: cluster size = 35 voxels, peak MNI coordinate *x*/*y*/*z* = 54/27/27, peak *t* = 4.79) of the ECN (Figure 6c), as well as a significant negative correlation between Shannon index and intranetwork connectivity in the right angular gyrus (AG) (cluster size = 30 voxels, peak MNI coordinate *x*/*y*/*z* = 36/−63/39, peak *t* = −4.26) of the rFPN (Figure 6d; *p* <.05, cluster‐level FWE corrected). However, there were no significant correlations between intranetwork functional connectivity and behavioral variables.

### 3.2 Associations between enterotypes, functional connectivity, and behaviors

**FIGURE 7:**
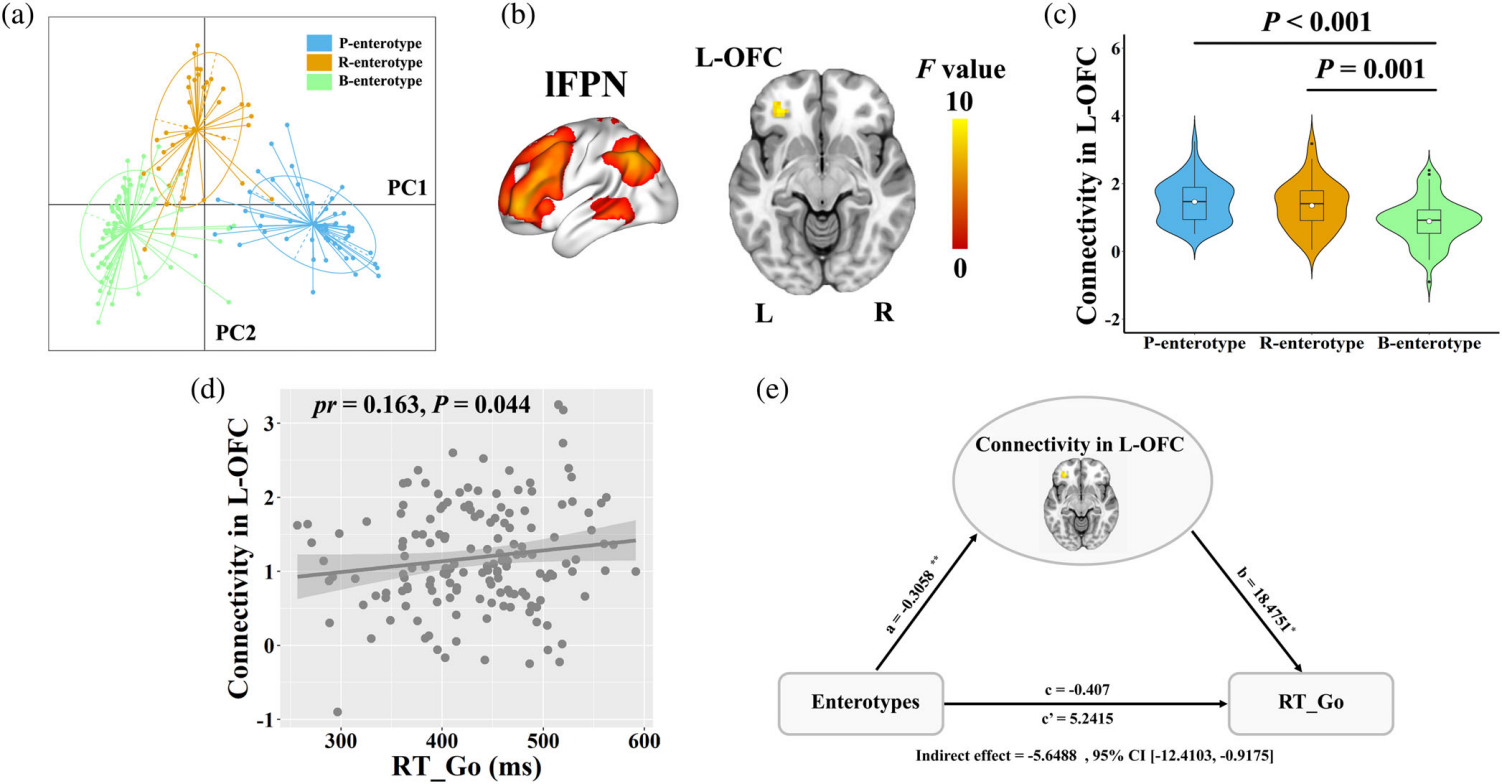
Associations between enterotypes, functional connectivity and behaviors. (a) All samples were clustered into three enterotypes. (b) Intranetwork functional connectivity in the left OFC of the lFPN differed across enterotypes. (c) The violin plot shows the distribution and between‐enterotype differences in intranetwork connectivity. (d) The scatter plot shows the correlation between intranetwork functional connectivity and RT‐Go. (e) The mediation analysis between enterotypes (X) and RT‐Go (Y), with intranetwork connectivity in the left OFC as the mediator. Path coefficients with *p* values (*p <.05 and **p <.01, respectively). B, bacteroides; L, left; lFPN, left frontoparietal network; OFC, orbitofrontal cortex; P, prevotella; PC, principal component; R, right; R, ruminococcaceae; RT‐Go, mean reaction time of correct responses in “Go” conditions

All samples were clustered into three well‐matched enterotypes (Figure 7a and Table 2). Prevotella, Ruminococcaceae, and Bacteroides genera were considered as enterotype identifiers (P‐, R‐, and B‐enterotypes) as they showed the largest variation in abundance, coinciding with prior studies (Arumugam et al., 2011; Falony et al., 2016; Vieira‐Silva et al., 2016).

**TABLE 2:** Demographic characteristics of the participants with three enterotypes

Characteristics	P‐enterotype	R‐enterotype	B‐enterotype	Statistics	*p* value
Number of subjects	51	37	69		
Gender (female/male)	24/27	21/16	32/37	*χ* ^*2*^ = 1.16	.561^a^
Age (years)	22.55 ± 2.39	21.78 ± 2.27	22.45 ± 2.49	*F* = 1.24	.291^b^
Education (years)	15.96 ± 1.93	15.54 ± 1.86	15.78 ± 1.96	*F* = 0.51	.602^b^
BMI (kg/m^2^)	21.76 ± 3.95	21.25 ± 2.47	21.30 ± 2.93	*F* = 0.39	.680^b^
FD (mm)	0.13 ± 0.07	0.12 ± 0.04	0.12 ± 0.04	*F* = 1.22	.299^b^

*Note*: All values are expressed as mean ± standard deviation.

Abbreviations: B, bacteroides; BMI, body mass index; FD, frame‐wise displacement; P, prevotella; R, ruminococcaceae.

^a^ The *p* value was obtained by Pearson Chi‐square test.

^b^ The *p* value was obtained by one‐way ANOVA.

One‐way ANOVA revealed significant differences in intranetwork connectivity in the left orbitofrontal cortex (OFC; cluster size = 26 voxels, peak MNI coordinate *x*/*y*/*z* = −33/39/−9, peak *F* = 9.58) of the lFPN across three enterotypes (Figure 7b; *p* <.05, cluster‐level FWE corrected). The *post hoc* pairwise comparisons demonstrated that participants with P‐ and R‐enterotypes showed increased intranetwork connectivity in the left OFC of the lFPN compared to those with B‐enterotype (Figure 7c). Correlation analysis with behaviors brought forward an observation of a significant positive correlation between RT_Go and intranetwork connectivity in the left OFC (*pr* = .163, *p* = .044) (Figure 7d). Further mediation analysis showed that intranetwork connectivity in the left OFC mediated the relationship between enterotypes and RT_Go (indirect effect = −5.6488, *SE* = 2.8203, 95% CI: −12.4103, −0.9175; Figure 7e). However, no significant differences in internetwork functional connectivity were observed across three enterotypes.

### 3.3 Sensitivity analysis

After additionally adjusting for BMI, our main results were preserved, that is, the correlations between alpha diversity and functional connectivity (Table S2) and the differences in intranetwork connectivity across enterotypes (*F* = 12.501, *p* <.001) remained unchanged. Likewise, by including DNHQ and IPAQ scores as additional nuisances, the correlations between alpha diversity and functional connectivity (Table S3) and the differences in intranetwork connectivity across enterotypes (*F* = 12.137, *p* <.001) remained unaltered, suggesting that dietary habit and physical exercise did not influence our results.

## 4 DISCUSSION

The present study opens new perspectives by being the first to assess the relationship among gut microbiome, large‐scale functional network connectivity and behaviors in a large sample of healthy young adults. We found significant associations of gut microbial diversity with internetwork functional connectivity between the executive control (ECN, lFPN, rFPN, and DAN), default mode (aDMN and pDMN), and sensorimotor (dSMN, AN, mVN, lVN, and pVN) systems, indicating widespread but nonspecific influences of microbial diversity on functional connectivity between large‐scale functional networks. Moreover, sleep quality, working memory and attention were related to some of the microbial diversity‐sensitive internetwork connectivity, which could serve as mediators of the associations between microbial diversity and these behaviors. In addition, gut microbial diversity showed correlations with intranetwork functional connectivity of the ECN and rFPN. Our data also demonstrated that compared to B‐, P‐, and R‐enterotypes exhibited increased intranetwork connectivity of the lFPN, which could mediate the association between enterotypes and executive function. These findings suggest a pronounced and specific effect of enterotypes on functional connectivity within the executive control system.

There have been prior efforts to examine the associations between gut microbiome and brain imaging measures in healthy and clinical conditions. Among them, only two resting‐state fMRI studies attempted to uncover the relationship between gut microbiome and functional connectivity of large‐scale brain networks. Reports of Gao et al. (2019) showed that gut microbial diversity was associated with functional connectivity of some canonical resting‐state functional networks in healthy infants. In that study, the construction of functional networks was based on a hypothesis‐driven seed‐based approach, which lacks a global and independent view because the seed regions must be specified a priori. This may introduce potential selection biases that might have influenced the results. This problem can be partially overcome by the data‐driven ICA approach, which automatically separates the signals of the whole brain into statistically independent components, resulting in spatially nonoverlapping functional networks (Damoiseaux et al., 2006; van de Ven et al., 2004). By means of the ICA approach, Wang et al. identified a link between gut microbiota and DMN topological measures in patients with end‐stage renal disease (Y. F. Wang et al., 2019). However, the investigators focused solely on DMN and place no emphasis on other functional networks. Thus, there is still a paucity of research investigating the effects of gut microbiota on different large‐scale functional networks in an unbiased fashion. In addition, converging evidence has established the relations between gut microbiota and multiple human behaviors including sleep quality, working memory, and attention (Arnoriaga‐Rodriguez et al., 2020; Cenit, Nuevo, Codoner‐Franch, Dinan, & Sanz, 2017; Grosicki, Riemann, Flatt, Valentino, & Lustgarten, 2020). In the current study, with the availability of fecal samples, resting‐state fMRI and a set of sleep and cognitive assessments comes the ability to further disentangle the gut microbiota‐functional network connectivity‐behavior relationships in young adulthood.

The executive control system, anchored mainly in the prefrontal and lateral parietal regions, is thought to be involved in a variety of cognitive‐control processes related to goal‐directed behaviors (Cole & Schneider, 2007; Xin & Lei, 2015), via complex interactions between its core components (intranetwork connectivity) and with other systems (internetwork connectivity). The observed strong effects of gut microbial diversity and enterotypes on inter‐ and intranetwork connectivity of the executive control system (ECN, lFPN, rFPN, and DAN) highlight its prominent involvement in the microbiota‐gut‐brain axis. Meanwhile, our data showed that functional connectivity of the default mode (aDMN and pDMN) and sensorimotor (dSMN, AN, mVN, lVN, and pVN) systems was also preferentially affected by the microbial diversity. The default mode system, primarily comprising medial prefrontal and medial parietal cortices, is implicated in a range of internally directed cognition such as emotional processing and self‐referential mental activity (Buckner, Andrews‐Hanna, & Schacter, 2008; Raichle, 2015). The sensorimotor system consists of widely distributed auditory, visual and sensorimotor cortices, which are engaged in sensory and motor processes. Our findings stand in accordance with previous studies that reported links between gut microbiota and resting‐state functional connectivity of the two systems (Curtis et al., 2019; Gao et al., 2019; Y. F. Wang et al., 2019).

The mediation analyses further revealed that functional connectivity of some large‐scale brain networks mediated the relations of gut microbiota with sleep quality, working memory, and attention. It is noteworthy that working memory and attention are core executive functions that may be influenced by sleep (Diamond, 2013), suggesting an intimate link between these behaviors. The current findings not only may add important context to the growing literature on the effects of gut microbiome on human behaviors by yielding insights into the potential neurobiological mechanisms underlying such effects, but also may be of high clinical relevance by exposing the gut microbiota as a promising target for treating and preventing brain disorders characterized by deficits in sleep or executive functions.

There are several limitations that should be mentioned. First, given that our study sample was selected from a group of educated subjects with a limited age range of 18–30 years, these findings might not be representative of the general population. Further research in participants with broader age and educational ranges may be warranted to validate our preliminary findings. The second limitation is the cross‐sectional nature of the design. Longitudinal work will be required to determine causal links between the gut microbiome and the brain. Third, although we identified meaningful functional networks from a range of ICA‐derived components according to a strict selection procedure, there are possible biases that might have influenced our interpretation. For example, some nontypical but physiologically relevant functional networks might have not been considered. Finally, we focused on gut microbial diversity and enterotypes because they are the most frequently used global parameters to characterize gut microbial community profiles. Other indices (e.g., relative abundance of the bacteria) derived from 16S analysis should be calculated to further examine the gut microbiota‐brain relationship in the future.

In conclusion, the results of this study provide first empirical evidence that the gut microbiota can modulate large‐scale inter‐ and intranetwork functional connectivity (especially the executive control system) in young adulthood. Moreover, some functional network connectivity may act as mediators of the effects of gut microbiota on sleep and executive functions. These findings might expand existing biological knowledge concerning the gut microbiota‐brain‐behavior relationships from the perspective of large‐scale functional network organization. More generally, they may ultimately inform a translational conceptualization of how to improve sleep quality and executive functions through the regulation of gut microbiota.

## Supporting information


**Table S1** Correlations between alpha diversity indices
**Table S2.** Correlations between alpha diversity and functional connectivity after additionally adjusting for BMI
**Table S3.** Correlations between alpha diversity and functional connectivity after additionally adjusting for DNHQ and IPAQ scores

## Data Availability

The data that support the findings of this study are available on request from the corresponding author. The data are not publicly available due to privacy or ethical restrictions.
