# Supplementary material for: Large‐scale functional network connectivity mediate the associations of gut microbiota with sleep quality and executive functions
Source: Hum Brain Mapp. 2021 Mar 19;42(10):3088–101. doi: 10.1002/hbm.25419 (PMC8193524; doi:10.1002/hbm.25419)
Supplement: Supplementary file 1 — Table S1 Correlations between alpha diversity indices Table S2. Correlations between alpha diversity and functional connectivity after additionally adjusting for BMI Table S3. Correlations between alpha diversity and functional connectivity after additionally adjusting for DNHQ and IPAQ scores [file HBM-42-3088-s001.doc]

**Supplementary Materials**

**Table S1.** Correlations between alpha diversity indices

|  | Sobs | Ace | Chao | Shannon | Simpson |
| --- | --- | --- | --- | --- | --- |
| Sobs | - | 0.957 | 0.966 | 0.488 | -0.284 |
| Ace | - | - | 0.980 | 0.399 | -0.226 |
| Chao | - | - | - | 0.423 | -0.240 |
| Shannon | - | - | - | - | -0.921 |
| Simpson | - | - | - | - | - |

**Table S2.** Correlations between alpha diversity and functional connectivity after additionally adjusting for BMI

|  | Sobs | | Ace | Chao | Shannon | Simpson |
| --- | --- | --- | --- | --- | --- | --- |
| Internetwork functional connectivity | | | | | | |
| aDMN-lFPN | | - | - | - | - | -0.198(0.014) |
| pDMN-rFPN | | - | - | - | - | 0.160(0.048) |
| pDMN-AN | | - | - | - | - | 0.195(0.016) |
| ECN-lVN | | - | - | - | - | -0.187(0.020) |
| lFPN-rFPN | | - | - | - | - | -0.214(0.008) |
| rFPN-DAN | | - | - | - | - | 0.253(0.002) |
| rFPN-dSMN | | - | - | - | - | 0.183(0.023) |
| rFPN-mVN | | - | - | - | - | 0.186(0.021) |
| rFPN-lVN | | - | - | - | - | 0.202(0.012) |
| DAN-pVN | | - | - | - | - | -0.212(0.008) |
| dSMN-pVN | | - | - | - | - | -0.164(0.043) |
| Intranetwork functional connectivity | | | | | | |
| L-LPFN of ECN | | 0.380(<0.001) | 0.401(<0.001) | 0.382(<0.001) | - | - |
| R-LPFN of ECN | | 0.372(<0.001) | 0.403(<0.001) | 0.369(<0.001) | - | - |
| R-AG of rFPN | | - | - | - | -0.320(<0.001) | - |

Abbreviations: BMI, body mass index; aDMN, anterior default mode network; lFPN, left frontoparietal network; pDMN, posterior default mode network; rFPN, right frontoparietal network; AN, auditory network; ECN, executive control network; lVN, lateral visual network; DAN, dorsal attention network; dSMN, dorsal sensorimotor network; mVN, medial visual network; pVN, posterior visual network; L, left; LPFC, lateral prefrontal cortex; R, right; AG, angular gyrus.

**Table S3.** Correlations between alpha diversity and functional connectivity after additionally adjusting for DNHQ and IPAQ scores

|  | Sobs | | Ace | Chao | Shannon | Simpson |
| --- | --- | --- | --- | --- | --- | --- |
| Internetwork functional connectivity | | | | | | |
| aDMN-lFPN | | - | - | - | - | -0.204(0.012) |
| pDMN-rFPN | | - | - | - | - | 0.199(0.014) |
| pDMN-AN | | - | - | - | - | 0.212(0.009) |
| ECN-lVN | | - | - | - | - | -0.196(0.015) |
| lFPN-rFPN | | - | - | - | - | -0.218(0.007) |
| rFPN-DAN | | - | - | - | - | 0.273(0.001) |
| rFPN-dSMN | | - | - | - | - | 0.213(0.009) |
| rFPN-mVN | | - | - | - | - | 0.243 (0.003) |
| rFPN-lVN | | - | - | - | - | 0.229(0.005) |
| DAN-pVN | | - | - | - | - | -0.237(0.003) |
| dSMN-pVN | | - | - | - | - | -0.210(0.009) |
| Intranetwork functional connectivity | | | | | | |
| L-LPFN of ECN | | 0.386(<0.001) | 0.405(<0.001) | 0.388(<0.001) | - | - |
| R-LPFN of ECN | | 0.375(<0.001) | 0.404(<0.001) | 0.370(<0.001) | - | - |
| R-AG of rFPN | | - | - | - | -0.362(<0.001) | - |

Abbreviations: DNHQ, Dietary Nutrition and Health Questionnaires; IPAQ, International Physical Activity Questionnaires; aDMN, anterior default mode network; lFPN, left frontoparietal network; pDMN, posterior default mode network; rFPN, right frontoparietal network; AN, auditory network; ECN, executive control network; lVN, lateral visual network; DAN, dorsal attention network; dSMN, dorsal sensorimotor network; mVN, medial visual network; pVN, posterior visual network; L, left; LPFC, lateral prefrontal cortex; R, right; AG, angular gyrus.
